# Post-orthodontic lower incisor inclination and gingival recession—a systematic review

**DOI:** 10.1186/s40510-018-0212-6

**Published:** 2018-06-18

**Authors:** Michele Tepedino, Lorenzo Franchi, Omar Fabbro, Claudio Chimenti

**Affiliations:** 1 Department of Surgery and Translational Medicine, Università degli Studi di Firenze, Firenze, Italy; 2 Department of Biotechnological and Applied Clinical Sciences, Università degli Studi dell’Aquila, Firenze, Italy

## Abstract

**Background:**

The present systematic review was carried out to determine the correlation between gingival recession/bone height and incisor inclination in non-growing post-orthodontic patients compared to adult untreated subjects or patients treated with different methodologies.

**Materials and methods:**

PubMed, EMBASE, Web of Science, Scopus, Cochrane, and OpenGrey databases were searched without time and language restriction. Search terms included orthodontic, incisor, inclination, angulation, proclination, and gingival. Articles involving human participants and adult subjects receiving orthodontic treatment with fixed appliance, having incisors position, bone height and/or gingival recessions evaluated pre- and post-treatment were included. Two authors independently extracted data using predefined forms. Risk of bias in individual studies was assessed with the Newcastle-Ottawa Scale.

**Results:**

Two observational studies were included in the qualitative analysis. The heterogeneity in outcome assessment among the studies did not allow performing a meta-analysis. The two studies, while observing some effects of orthodontic treatment on the development of gingival recession, reported that these effects were not statistically or clinically significant.

**Conclusions:**

There is no strong scientific evidence concluding that proclination of incisors by means of fixed orthodontic appliances can affect periodontal health. Further prospective studies are required to elucidate this statement.

**Protocol:**

PROSPERO database registration number CRD42016042369.

## Background

One of the key points of orthodontic treatment planning is the position of the upper and lower incisors. Several authors have [1–3] described this as an important determinant for the patient’s facial appearance. Indeed, the position and esthetics of the lips can be influenced by that of the incisors, and this should be taken into account when establishing the treatment objectives. In addition, while determining the final position of the incisors, an excessive incisor proclination should be avoided for the risk of moving the teeth out of the alveolar envelope and developing a bone dehiscence, thus creating the risk of gingival recession [4]. Expanding the arches and proclining teeth is a viable alternative to extractions for space recovery. Many authors, however, observed that the position of the incisors into the alveolar bone can influence the gingival attachment [5–7] and long-term stability [8].

Despite these recommendations, there is a lack of scientific consensus and contradicting results that can be found in the literature [9]: Renkema and colleagues found that proclination of lower incisors did not increase the risk of gingival recession 5-year post-treatment in adolescents [10], and similarly, a systematic review concluded that no evidence can be found to confirm that proclination of lower incisors really affects the development of gingival recessions [11]. On the other hand, some studies on adolescent patients or mixed-age samples reported an increased risk of gingival recessions [12, 13]. Vassalli et al. [14] in their systematic review that included both animal and human studies concluded that a cause-effect relationship could be present but highlighted that this was supported by a low level of evidence. Because these two systematic reviews [11, 14] included also studies on children and adolescents that could have been biased by vertical growth of the alveolar processes, and new studies were published recently, it was decided to perform a new systematic review of the literature.

The present systematic review, therefore, was carried out to answer the following PICOS (Patients, Intervention, Comparator, Outcome, and Study design) question: to determine the correlation between gingival recession/bone height and incisor inclination in non-growing post-orthodontic patients compared to adult untreated subjects or patients treated with different methodologies, by measuring the clinical crown length or assessing the presence of gingival recession and/or alveolar bone height. The answer to this question could provide useful information to the clinician regarding treatment options and biomechanical management.

## Materials and methods

### Protocol

This systematic review was conducted according to the guidelines of the Cochrane Handbook for Systematic Reviews of Interventions and is reported following the PRISMA statement [15, 16]. Methods of the analysis and inclusion criteria were specified in advance and documented in a protocol registered in the National Institute of Health Research database (http://www.crd.york.ac.uk/PROSPERO Protocol: CRD42016042369). No funding was given for the realization of the present review.

### Eligibility criteria

Randomized and non-randomized prospective, retrospective, and observational original studies with the following characteristics, reported according to the PICOS format, were included: all types of human studies (studies), on adult patients undergoing orthodontic treatment (population) with fixed multibracket appliance (intervention), reporting post-treatment development of gingival recession and/or bone height measurements, and post-treatment position of incisors (outcome), compared to adult untreated subjects or patients treated with different methodologies (comparator).

It was decided to include also non-randomized studies based on the recommendations of the *Cochrane Handbook for Systematic Reviews of Interventions* [17]: the reasons for including non-randomized studies in a systematic review are (a) high-quality non-randomized studies could produce a better unbiased effect size compared with low-quality randomized controlled trials; (b) randomized controlled trials could be unavailable for ethical reasons; (c) non-randomized studies offer a perspective of the validity of the current literature and show the need for future research; (d) findings of a review of non-randomized studies might be useful in designing future studies; and (e) non-randomized studies could reveal potential unexpected, rare, or long-term harms of interventions [17, 18]. Case reports, case series, letters, opinion articles, conference abstracts, reviews, meta-analysis, and animal studies were excluded, as well as studies involving growing subjects and patients treated with functional appliances or orthognathic surgery and studies not reporting post-treatment gingival recessions, bone height, or incisor position as their primary and secondary outcomes.

### Information sources and search

The following databases were searched in June 2017, without language and initial date restrictions: MEDLINE via PubMed, EMBASE, Scopus, Web of Science, and Cochrane Library. Gray literature was searched in the OpenGrey database, without language and initial date restriction, up to June 2017. The search strategy for PubMed, which was appropriately adapted for each electronic database consulted, was the following: (orthodontic OR orthodontics) AND incisor AND (inclination OR angulation OR proclination OR gingival). In addition, manual search of the reference list of the potential studies was performed to retrieve additional articles. Duplicate articles were removed.

### Study selection

**Fig. 1:**
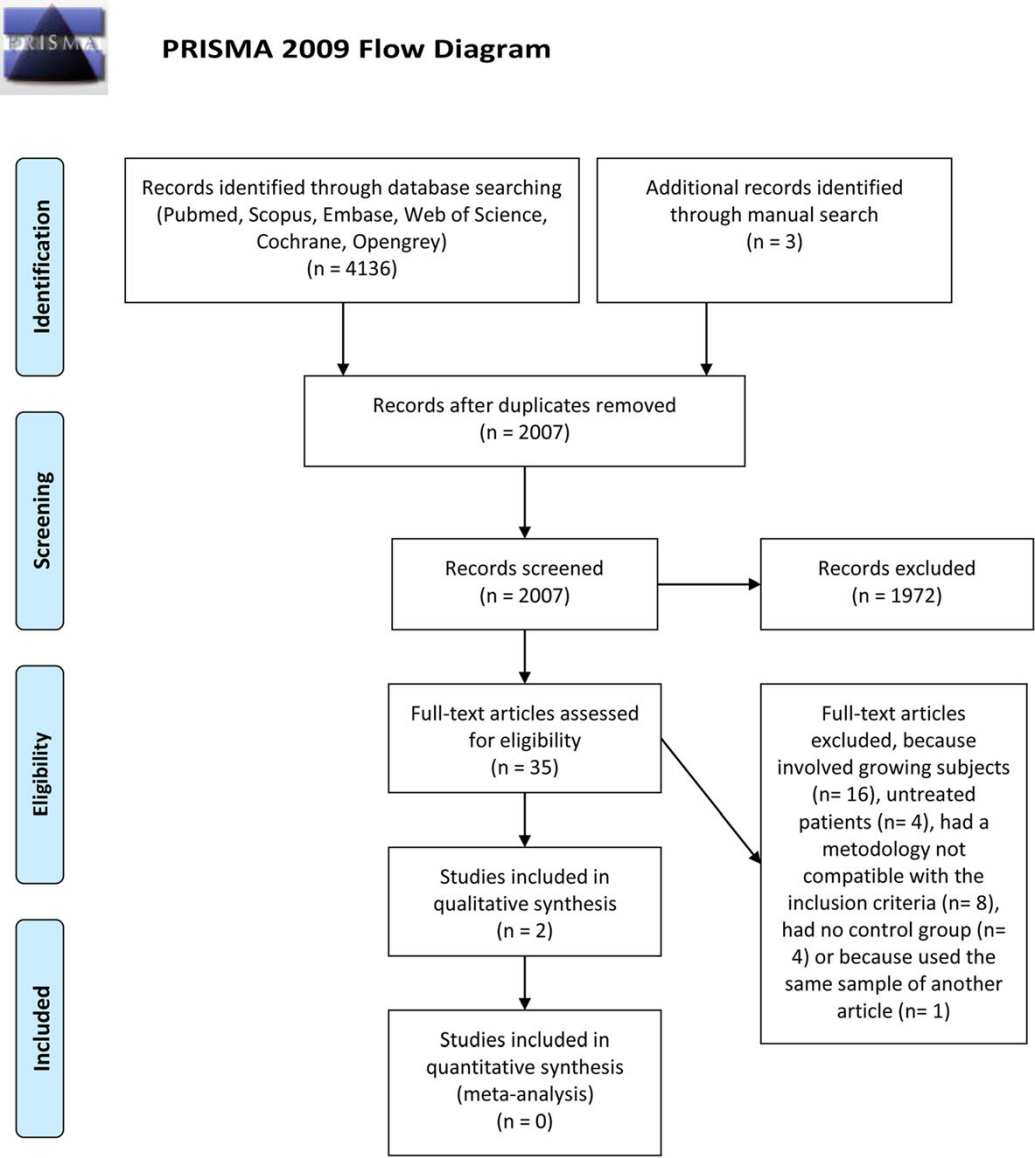
PRISMA flow diagram

Eligibility was assessed independently by two authors (MT and OF), screening initially title and abstract of the articles. Full texts were accessed whenever it was not clear if the abstract should be included or not. If the full text did not provide the necessary information, an attempt was made to contact the author by e-mail. Any disagreement was resolved by discussion and consensus or by a third experienced author who was requested to arbitrate (LF). PRISMA flowchart diagram for the study selection process is reported in Fig. 1. Inter-rater agreement between the independent assessments of the two reviewers (MT and OF), before reaching a consensus, was calculated using Cohen’s kappa statistics.

### Data collection

Two authors (MT and OF) independently extracted data (authors and year of publication, study design, sample size, sample composition by sex and age, type of orthodontic treatment, follow-up period, primary outcome and its method of assessment, secondary outcome and its method of assessment, results for primary and secondary outcomes) from the selected studies using a pre-determined extraction form. Disagreement between the two authors was resolved by discussion and consensus. If data were not sufficiently clear, the authors were contacted by e-mails. Care was taken to avoid inclusion of duplicated data from different articles.

### Risk of bias in individual studies

To assess the risk of bias in the studies finally included in the analysis, the Newcastle-Ottawa Scale [19] was applied independently by two reviewers (MT and OF). This scale is specially designed for observational studies and has nine domains, and each one can earn a “star” if judged to be satisfactory in the study. Up to four stars can be assigned for the domains regarding patient selection, up to two stars can be assigned for domains concerning comparability of the patient groups, and up to three stars for outcome assessment. Any disagreements between the two authors were solved by the intervention of a third experienced reviewer (LF).

### Summary measures and approach to synthesis

Due to the heterogeneity among the studies included in this systematic review, particularly in the variables used to evaluate gingival recessions and bone height, it was not possible to perform a meta-analysis. A narrative synthesis was performed by illustrating the results from individual studies according to the groups evaluated.

## Results

### Study selection

Electronic database search provided a total of 4136 results. Three additional articles were retrieved through manual search. After adjusting for duplicates, 2007 entries were left. Of these, 1886 titles were discarded because these were clearly not relevant, while 121 abstracts were screened. Eighty-six abstracts were discarded due to methodology not corresponding to the inclusion criteria or because growing patients were involved, and then, 35 full-text papers were accessed for detailed examination, comprising one article in Chinese, one in Polish, and one in German language. Two studies [20, 21] were conducted on the same sample of patients; therefore, only the first was enclosed in the systematic review. Two studies were finally included in the systematic review (Fig. 1).

Inter-rater agreement between the reviewers was good, with Cohen’s kappa having a value of 0.857 (*p* < 0.001).

### Study characteristics

The two studies finally included in the qualitative analysis (Table 1) were all retrospective observational studies. One study [22] did not report gender distribution of the samples: the authors were contacted by email to try to retrieve this information, without success. Both the two studies had a control group: one using untreated patients [20] and the other comparing extraction vs non-extraction treatment cases [22]. Regarding the type of orthodontic intervention, both studies used straight wire fixed orthodontic appliances, one with extractions [22]. The article by Villard and Patcas [22] was the only one reporting also a follow-up examination 3 years post-treatment.

**Table 1:** Characteristics of the studies included in the qualitative analysis

Author	Publication year	Language	Study design	Sample size	Control group	Type of patients and/or subgroups	Mean age	Sex distribution (M/F)	Follow-up time	Details on ortho treatment	Primary outcome measure	Method of assessment for primary outcome	Secondary outcome	Method of assessment for secondary outcome	Primary outcome result	Secondary outcome result
Allais and Melsen [20]	2003	English	Retrospective	150	Yes	Adult	33 years (22–65)	36/114	Debonding	Fixed appliance with proclination	Recession (mm), plaque score, gingival index, biotype	Dental casts and photo	Incisor proclination	Arch length changes on dental casts, IMPA on cephalograms	Incidence of recession is higher for treated than that for control for teeth 41 and 32, not 42 and 31 (differences within the method error)	Mean arch length increase of 3.4 ± 2.6 mm
Villard and Patcas [22]	2015	English	retrospective	50	Yes	Adult with or without premolar extractions	23.8 ± 2.8 extraction group; 24.8 ± 6.1 non-extraction group	N.S.	3 years after debonding	Fixed appliance, 24 extraction and 26 non-extraction	Crowding, clinical crown length	casts	IMPA, distance of L1 and B from perpendicular to MP passing through Pg	cephalograms	Clinical crown length increase of 0.4–0.8 mm in non-extraction group and of 1.1–0.4 in extraction group	Weak correlation between incisor position and clinical crown length, no correlation for canines

*N.S.* not specified

There was a large heterogeneity among the included studies in assessing gingival recessions, with one study evaluating gingival recession on casts and the other on intraoral photographs. Concerning post-treatment incisor position, one study [22] used the incisor–mandibular plane angle (IMPA) as a measure, while in the other [20], the change in incisor position was assessed by arch length changes.

### Risk of bias in individual studies

Assessment of risk of bias by means of the Newcastle-Ottawa Scale is reported in Table 2. All the two included studies earned more than seven “stars,” but no one achieved the maximum score.

**Table 2:** Assessment of risk of bias of observational studies with the Newcastle-Ottawa Scale

		Allais and Melsen [20]	Villard and Patcas [22]
Design		Cohort study	Cohort study
Selection	Representativeness of the study group	*	*
	Selection of the controls	*	*
	Ascertainment of exposure	*	*
	Definition of baseline characteristics		*
Comparability	Comparability for gingival recessions	*	*
	Comparability for incisor inclination	*	
Outcome	Assessment of outcome	*	*
	Adequacy of follow-up		*
	Attrition during follow-up	*	*
Total		7	8

### Qualitative synthesis

Allais and Melsen [20] retrospectively retrieved a sample of 150 adult patients with class I and II malocclusion treated with fixed appliances and a control group of 150 patients waiting for orthodontic treatment. Sample size was determined by power analysis, and cases and controls were pair-matched by sex and age by a simple random sampling. The authors assessed visual plaque, gingival inflammation, and gingival biotype on color slides; gingival recessions were assessed by a blinded operator both on color slides and dental casts measuring the distance between the gingival margin and the cemento-enamel junction (CEJ). Lateral cephalograms were used to measure at baseline the angulation of the axis of the lower incisor with respect to the mandibular plane (IMPA) and the distance in millimeters between the edge of the lower incisor and the A-Po line; in addition, the change in arch length following incisor proclination was measured on dental casts. In the treatment group, incisor proclination determined a mean arch length change of 3.4 mm (SD 2.6 mm). This finding, according to the authors, affected gingival health: 35% of treated cases had at least one incisor with gingival recession, compared to 17% of controls, and this difference is being statistically significant (*p* < 0.05). The authors found that the occurrence of recessions was significantly higher in treated patients compared to that in controls for lower right central incisor and lower left lateral incisor, but not for lower left central incisor and lower right lateral incisor. All differences, however, were within the error of the method.

Villard and Patcas [22] retrospectively selected 50 adult patients having class I malocclusion, moderate crowding in the lower jaw, and gingival and periodontal health of all teeth. Of these patients, 24 were treated with fixed appliance and extractions of first premolars in the lower jaw, while 26 were treated without extractions, thus defining two different groups. The authors used dental casts to measure Little’s irregularity Index [23] and clinical crown length of the lower incisors and canines and cephalometric tracings to calculate the IMPA angle. All these measurements were performed pre-treatment, post-treatment, and 3 years after debonding. Because extractive therapy hardly differs from non-extractive treatment, only measurements from the non-extraction group were used, to have data comparable with those of the study from Allais and Melsen. After treatment, a mean change of clinical crown length of 0.84 ± 0.9 mm was observed for the canines and 0.37 ± 0.7 mm for the incisors. A statistically significant (*p* = 0.027) correlation between clinical crown length of the lower incisors and their inclination was found, but the coefficient of this correlation was very weak (*ρ* = 0.158). Therefore, no definitive conclusions can be drawn from such data. In addition, the regression analysis performed by the authors was not able to detect any significant predictor of post-treatment recessions.

## Discussion

### Summary of evidence

The study by Allais and Melsen [20] was included also in the previous systematic reviews, while the other [22] was published subsequently and never enclosed in a systematic review. Overall, the evidence is not sufficiently robust to determine an influence of post-orthodontic treatment position of lower incisors and incidence of gingival recessions. No randomized clinical trials were found during the literature search, and the studies finally included in the review were all retrospective. Indeed, it is difficult to design a randomized controlled trial to study the effects of incisor’s proclination during orthodontic treatment: it would be difficult and ethically questionable to randomize the treatment modalities and deliberately procline teeth in some patients, or to perform stripping or extractions in others, while teeth movements will always be a choice that follows clinician’s assessment. Moreover, many factors can participate alone or together with others to the pathogenesis of gingival recessions and can be difficult to control, like oral hygiene, brushing habits, and smoking; gingival recessions may occur several years after treatment; thus, a long observation time is needed. In such case, therefore, non-randomized studies may have an important role in providing evidence and defining the boundaries for future high quality trials [17, 18, 24], so we decided to perform a systematic review on this type of studies.

It was not possible to carry out a meta-analysis due to the heterogeneity of the methods among the included studies: there were different types of orthodontic treatment, and the presence of gingival recession was assessed with different methodologies; therefore, data were not comparable.

Allais and Melsen conducted a very well-designed retrospective study [20] that included a control group. Although they found a statistically significant effect of lower incisor’s proclination on gingival health, this effect regarded only the lower right central incisor and the lower left lateral incisor. Moreover, this finding was not significant from a clinical point of view, because the magnitude of the difference between treated patients and controls was very small (by mean 0.14 mm) and within the range of the calculated standard deviation of the error of the method.

The work by Villard and Patcas [22] earned the highest score in the risk of bias assessment. The authors considered patients treated with extractions and retraction of lower incisors, as a control for patients treated without extractions and with proclination of the lower incisors. Considering only data from the non-extraction group, they observed an increase in clinical crown length post-treatment that was higher for canines than incisors. However, no correlation was found between the final inclination of lower teeth and gingival recession, nor the authors were able to observe any factor that could serve as a predictor of development of gingival recession. In addition, the use of dental casts to measure clinical crown length can be questionable.

### Limitations

It must be underlined again that the included studies were retrospective studies and therefore could be affected by selection bias [25], and some confounders could have not been adequately controlled, like the patient’s oral hygiene and habits. This can be considered the main limitation of the present systematic review.

It was decided to include only studies performed on adult patients, to exclude growth as a possible confounding factor. Dental casts or intraoral photographs are not the best methods to measure gingival recessions, however were the only possible for a retrospective study.

Summarizing these results, there is no consensus or strong scientific evidence to demonstrate that proclination of the lower incisors as a consequence of orthodontic treatment can lead to gingival recession. High-quality prospective studies, with clear definition and standardization of orthodontic treatment modalities and possible use of three-dimensional radiographs, should be designed to provide scientific evidence for a topic that is surely important for everyday clinical practice.

## Conclusions

Based on the results of the present systematic review, it is possible to conclude the following:

1. No scientific evidence exists stating that proclination of incisors following orthodontic treatment with fixed appliance increases the risk of gingival recession.
2. Further prospective or randomized studies are needed to clarify to which extent proclination of incisors should be considered a risk for periodontal health.

## Abbreviations

CEJ: Cemento-enamel junction
IMPA: Incisor–mandibular plane angle
PICOS: Patient, Intervention, Comparator, Outcome, and Study design
PRISMA: Preferred Reporting Items for Systematic Reviews and Meta-Analyses
